# Leveraging Global Fund’s investments to expand innovative public-private provider engagement in TB

**DOI:** 10.5588/ijtldopen.24.0162

**Published:** 2024-06-01

**Authors:** M.A. Yassin, L. Kimbo, E. Wandwalo, A. Rashid, A. Dzokoto, U. Weber, G. Stallworthy

**Affiliations:** ^1^ Global Fund to Fight AIDS, Tuberculosis and Malaria, Geneva, Switzerland,; 2 The Bill and Melinda Gates Foundation, Seattle, USA

**Keywords:** tuberculosis, public-private mix, notification, Global Fund, innovation

## Abstract

**BACKGROUND:**

TB remains a significant global health threat, claiming 1.3 million lives annually. The COVID-19 pandemic disrupted progress in the global TB response. Most patients with TB initially seek care from private providers, whereas only a small proportion are engaged by national programmes. The Global Fund is the major international source of funding for TB responses and supports the scale-up of innovative private-public mix (PPM) models in TB.

**METHODS:**

We collected programmatic and financial data on TB from 11 priority countries implementing PPM activities**.** Country examples and trends in the budget of Global Fund grants were analysed.

**RESULTS:**

These countries account for 60% of the global TB burden and Global Fund TB portfolio. PPM contributed 29% of national TB notifications in 2022 (range: 8% to 49%). During 2021–2023, US$1.4 billion was allocated for TB and US$155 million (11%) for PPM, while PPM contributed to 35% of national TB notification targets. PPM budgets increased over time from US$43 million (2002 to 2014) to US$129 million (2024 to 2026).

**CONCLUSION:**

The Global Fund’s investments facilitated the expansion of innovative PPM models, improved access, and enhanced TB responses. Our indicative analysis underscores the need for evidence-based planning, collaboration, and increased domestic investment to accelerate the end of TB.

## RÉSUMÉ

**CONTEXTE:**

La TB reste une menace importante pour la santé mondiale, faisant 1,3 million de morts chaque année. La pandémie de COVID-19 a perturbé les progrès de la riposte mondiale à la TB. La plupart des patients atteints de TB recherchent d'abord des soins auprès de prestataires privés, tandis que seule une petite proportion est engagée par des programmes nationaux. Le Fonds mondial est la principale source internationale de financement de la lutte contre la TB et soutient l'extension de modèles innovants de partenariats public-privé (PPM, pour l’anglais « *public-private mix* ») dans le domaine de la TB.

**MÉTHODES:**

Nous avons recueilli des données programmatiques et financières sur la TB dans 11 pays prioritaires mettant en œuvre des activités de PPM. Des exemples de pays et les tendances du budget des subventions du Fonds mondial ont été analysés.

**RÉSULTATS:**

Ces pays représentent 60% de la charge mondiale de morbidité de la TB et du portefeuille du Fonds mondial de lutte contre la TB. La PPM a contribué à 29% des notifications nationales de TB en 2022 (fourchette : 8–49%). Au cours de la période 2021–2023, 1,4 milliard de dollars US ont été alloués à la TB et 155 millions de dollars US (11%) à la PPM, tandis que la PPM a contribué à 35% des cibles nationales de notification de la TB. Les budgets PPM ont augmenté au fil du temps, passant de 43 millions de dollars US (2002 à 2014) à 129 millions de dollars américains (2024 à 2026).

**CONCLUSION:**

Les investissements du Fonds mondial ont facilité l'expansion de modèles PPM innovants, l'amélioration de l'accès et le renforcement des ripostes à la TB. Notre analyse indicative souligne la nécessité d'une planification fondée sur des données probantes, d'une collaboration et d'une augmentation des investissements nationaux pour accélérer l'éradication de la TB.

TB remains a major infectious disease killer, claiming approximately 1.3 million lives in 2022 alone.^1^ Response to TB has been gaining momentum with a reduction in incidence and mortality in recent years. However, the COVID-19 pandemic disproportionately affected the global TB response, leading to a decrease in the number of people diagnosed and treated for TB, including drug-resistant TB (DR-TB),^1^ leading to ongoing transmission, increased incidence, and mortality.

Global efforts to end TB require urgent and concerted attention, new tools and innovative solutions, and additional resources. Most of the missing people with TB access services through private healthcare providers, who often serve as the initial point of care and consultation. This is particularly true in many countries in Asia (Bangladesh, India, Indonesia, Myanmar, Pakistan and the Philippines) and in Nigeria, where an average of 79% (67–90%) of initial consultations occur in the private sector.^2–4^

Engaging private healthcare providers is crucial to improving access and quality of care and accelerating progress towards ending TB. The presence of unregulated and informal providers, perceptions about the type and quality of service provided, and the lack of prioritisation and resource allocation challenge their engagement, although there have been improvements over time.^5^ Countries have been implementing public-private mix (PPM) models to engage private providers in the screening, referral, diagnosis and treatment of people with TB. To improve notification and quality of care, national TB programmes (NTPs) collaborate with partners to provide technical support and guidance, training, access to diagnostic testing, drugs and reporting tools for private providers.^5^

The WHO and partners have developed a PPM Roadmap for TB prevention and care, which outlines 10 key actions. These include understanding patients’ preferences and private sector dynamics, establishing supportive policies, setting ambitious targets, adapting flexible engagement models, advocating for political commitment and investment in PPM, leveraging digital technologies, allocating adequate funding, delivering a range of incentives, partnering with stakeholders, and monitoring progress.^3^

The Global Fund is the main source of international funding for TB, accounting for over 75% of external (non-domestic) funding in low- and middle-income countries.^1^ As a unique public-private partnership, the Global Fund encourages private sector engagement to accelerate TB responses. The Global Fund has been providing additional funding to support PPM activities as part of its catalytic/matching funding to find missing people with TB since 2017. Additional funding for TB was leveraged through the Global Fund’s COVID-19 Response Mechanism (C19RM), which supported countries to mitigate the impact of COVID-19 on TB and accelerate recovery in TB response. In 2022, there was a notable surge in TB screening and testing globally, with 6.7 million people diagnosed and treated with TB, surpassing the 5.8 million reported in 2019.^6^

Following a successful replenishment, the Global Fund has allocated over US$13 billion for HIV, TB, and malaria and for building resilient and sustainable health systems during the 7th Grant Cycle (GC7: 2024–2026).^7^ In 2023, most countries submitted funding requests and completed the grant-making processes to prepare for implementation in 2024.

The aim of our study was to assess the Global Fund’s investments in PPM activities and the contributions of private healthcare providers to TB response in countries with a high burden of TB to promote the sharing of lessons learned and encourage adaptation and scale-up of successful models.

## METHODS

This is a retrospective study in which we collected TB programmatic and financial data from selected priority countries that receive more than half of the Global Fund TB funding and have implemented PPM activities during Grant Cycle 6 (GC6: 2021–2023). Data on the Global Fund’s allocation for TB, total budget, budget allocated for PPM, and performance (results compared to targets) were collected from the Global Fund’s grant documents, progress updates, performance framework, funding requests, and data on TB notification from the WHO database. Because our study is based on retrospective data, ethical approval was not required.

Additional information was collected through questionnaires sent to the Global Fund’s country teams and countries. The information collected through questionnaires included grant implementation arrangements, funding recipients (principal recipients [PRs] are entities that directly receive funding from Global Fund and contracted to manage grants, implement agreed-upon activities and deliver results and sub-recipients [SRs] are contracted by PRs to receive funding and execute a set of agreed-upon activities), and the types of private providers (including private hospitals, health centres, clinics, practitioners, pharmacies, drug stores, private laboratories, and others) engaged during GC6.

The inclusion criteria for the analysis were a TB budget of at least US$45 million and a PPM budget of at least US$1.5 million in GC6. Data were collected for 11 countries (Bangladesh, Ethiopia, India, Indonesia, Kenya, Myanmar, Nigeria, Pakistan, Philippines, Tanzania and Vietnam) that met the criteria. A trend analysis was conducted for seven countries (Bangladesh, India, Indonesia, Myanmar, Nigeria, Pakistan and Philippines) which have data on total TB funding and budget for PPM from the Global Fund between 2002 and 2026. Country experiences implementing and scaling up PPM activities were collected from a subset of countries through desk reviews and country missions.

## RESULTS

In 2021, the 11 countries collectively contributed to more than 60% of the worldwide burden of TB, and a total of 3,963,569 TB notifications, achieving 79% of the Global Fund performance framework target, with percentages ranging from 46% in Myanmar to 105% in Bangladesh and 133% in Nigeria. In 2022, these countries notified a total of 4,939,414 TB cases, reaching 98% of the target. The percentages varied from 73% in Vietnam to 102% in Ethiopia and Tanzania, with Nigeria exceeding the target at 146% (Table 1). In 2021, only Bangladesh, Nigeria, and Tanzania achieved over 95% of the notification targets as the other countries were still recovering from the impact of COVID-19. However, by 2022, all 11 countries either reached or surpassed pre-COVD-19 notification levels, with almost a third of notifications being contributed by PPM. On average, PPM contributed 29% and 28% of national notifications in 2021 and 2022, respectively, ranging from 8% in Vietnam to 49% in Myanmar. Overall, 1,149,273 and 1,399,612 people with TB were notified through PPM in 2021 and 2022, respectively (an increase of 250,399 TB notifications in 2022 compared to 2021).

**Table 1.** TB notifications and the contributions of PPM to notifications in 2021 and 2022.

Country	2021					2022				
	Notification target*	Notification result*	Achievement %^†^	PPM contribution to notification result	Proportion of PPM contribution %^‡^	Notification target*	Notification result*	Achievement %^†^	PPM contribution to notification result	Proportion of PPM contribution %^‡^
Bangladesh	292,746	306,536	105	70,979	23	298,112	261,957	88	67,583	26
Ethiopia	125,122	104,118	83	18,611	18	123,525	125,945	102	23,138	18
India	2,340,000	1,941,928	83	644,864	33	2,250,000	2,255,000	100	697,892	31
Indonesia	726,752	398,086	55	76,723	19	768,882	741,794	96	146,605	20
Kenya	101,323	77,921	77	15,353	20	99,910	91,087	91	16,913	19
Myanmar	142,893	65,801	46	17,112	26	139,510	107,086	77	52,117	49
Nigeria	156,346	207,895	133	58,420	28	195,432	285,563	146	69,471	24
Pakistan	422,462	373,370	88	155,726	42	440,939	424,560	96	178,271	42
Philippines	470,604	321,564	68	70,006	22	493,326	445,726	90	122,732	28
Tanzania	90,146	87,415	97	14,852	17	97,446	99,539	102	15,120	15
Vietnam	121,000	78,935	65	6,627	8	139,000	101,159	73	9,770	10
Total	4,989,394	3,963,569	79	1,149,273	29	5,046,082	4,939,416	98	1,399,612	28

* Targets and results are part of the Global Fund Performance Framework and are national.

† Calculated using the formula: notification results/notification targets*100.

‡ Calculated using the formula: PPM contribution to notification result/overall notification result*100.

PPM = private-public mix.

All 11 countries have legislation on mandatory notification, and TB is a notifiable disease. However, only four countries have implemented legally binding measures. For example, in the Philippines, the government has implemented mandatory TB notification that requires public and private healthcare providers to report patients diagnosed with TB. This initiative was supported by the deployment of Mandatory Notification Officers and contributed to an increase in TB notifications from private providers.^8,9^

Table 2 shows a summary of the number of PRs and SRs supporting the engagement of private providers on TB and the number of private providers/implementers engaged. Eighteen PRs and 124 SRs have supported PPM activities across 11 countries. Some PRs and SRs support PPM activities exclusively, while others also support part of NTPs’ public sector activities. A total of 251,951 private healthcare providers and facilities were engaged, with India, Nigeria, and the Philippines having the highest number of providers engaged in GC6. In all 11 countries, private providers are engaged in providing TB services along the cascade of care, including referral, diagnosis, treatment, and prevention, although their engagement has been on notification.

**Table 2.** Implementation arrangement and implementers/service providers* (Grant Cycle 6: 2021–2023).

Country	Principal recipients supporting PPM	Sub-recipients supporting PPM	Private hospitals, polyclinics, health centres	Private practitioners (individual, group)	Pharmacies, drug stores, dispensaries	Private laboratories	Other
Bangladesh	2	24	66	12,168	9,000	710	36
Ethiopia	1	10	1,199				
India	3	9	40,473	3,486	105,002	5,898	
Indonesia	2	19	1,359	165			
Kenya	2	35	336		146		
Myanmar	2	3	36	1,433	1,429		25
Nigeria	1	4	3,759		22,001	399	
Pakistan	1	8	295	7,402	4,511	680	8
Philippines	1	1	3,518	11,392		90	
Tanzania	2	2	521		4,180		5
Vietnam	1	9	1,525	2,701	6,668		5
Total	18	124	53,087	38,747	152,937	7,097	71

* Private healthcare providers engaged by the NTPs (as per the national policy) through PPM, regardless of whether they report TB cases during the specific period.

PPM = private-public mix; NTP = national TB programme.

The total amount of TB grants from the Global Fund for the 11 countries during GC6 was US$1.4 billion, which is higher than the allocation during GC5 and GC4, accounting for over 60% of the total Global Fund funding for TB. The average share of the budget for PPM was 11% (ranging from 3% in Ethiopia and India to 25% in Myanmar), while the contribution of PPM to national TB notification targets was 35% (ranging from 5% in Vietnam, 36% in Pakistan and 42% in India) (Table 3). The proportion of PPM contribution to national notification was higher than the proportion of PPM budget in all countries except Vietnam. In Myanmar, there was a good correlation between the two proportions, whereas there were wide variations in the other countries. We provide examples of private sector engagement activities in select countries, designed/adopted according to their contexts and regulatory environment as follows:

**Table 3.** Total TB grant, budget for PPM and contributions of PPM to national TB notification targets, 2021–2023.

Country	Total TB grant/budget (USD)	Budget for PPM (USD)	PPM budget %	TB notification target	PPM contribution to notification target	PPM contribution to target %
Bangladesh	121,767,021	15,615,893	13	894,117	219,960	25
Ethiopia	56,893,736	1,774,351	3	366,422	76,751	21
India*	273,582,332	7,582,474	3	6,750,000	2,824,239	42
Indonesia	170,000,000	14,096,111	8	2,264,005	725,314	32
Kenya	64,694,297	3,547,036	6	299,774	83,890	28
Myanmar	99,126,255	24,998,046	25	418,305	96,441	23
Nigeria	155,561,432	32,457,267	21	609,178	213,212	35
Pakistan	148,048,745	30,526,365	21	1,325,429	481,020	36
Philippines	171,920,126	9,915,062	6	1,471,238	417,841	28
Tanzania	49,088,020	7,262,213	15	286,655	85,997	30
Vietnam	69,884,327	7,531,332	11	398,000	20,070	5
Total	1,380,566,291	155,306,150	11	15,083,123	5,244,735	35

* India’s PPM activities (Patient Provider Support Agency) were transitioned to domestic funding during the current grant cycle with reduction in the budget.

PPM = private-public mix.

Bangladesh has a rich history of actively engaging with both for-profit and not-for-profit private healthcare providers. In 2021, these providers played a substantial role, contributing 24% of total TB notifications. Key initiatives include engaging graduate private practitioners and non-graduate private practitioners and establishing ‘DOTS Corners’ in hospitals, private medical colleges and workplace programmes. The private sector PR established 62 TB Diagnostic Centres equipped with digital X-rays and GeneXpert machines (Cepheid, Sunnyvale, CA, USA), providing free screening/testing services for people with presumptive TB. In addition, the NTP contracts with the private sector SR to offer chest X-ray and rapid molecular diagnostics services to patients referred by community health workers and the private sector. Private providers and communities contributed to a quick recovery in TB notifications in late 2020. All TB patients receive treatment at nearby DOTS centres, supported by community health workers. Bangladesh maintains an impressive treatment success rate of over 95%. This comprehensive approach demonstrates effective private sector engagement, significantly contributing to TB response in Bangladesh.

India exemplifies the evolution of private sector engagement in the TB response. The initial model was led by the National TB Elimination Programme (NTEP) and funded by the Bill and Melinda Gates Foundation (Seattle, WA, USA), which was expanded with support from the Global Fund. Between 2018 and 2020, the JEET (Joint Effort for Elimination of TB), further scaled up the PPSA (Patient-Provider Support Agency) model in over 100 districts in nine high TB burden states. This model, facilitated by a central, private sector technical support unit and state-based interface agencies, ensures comprehensive access to TB diagnosis, treatment, and patient support. Transitioning to domestic funding between 2021 and 2022, the PPSA model was scaled up by the NTEP via additional support from a World Bank loan, with financial contributions from the Global Fund. Notably, the private sector contributed significantly to TB notifications, with nearly a third of the 2.4 million patients with TB notified nationally in 2022.

Nigeria’s PPM approach to TB management is characterised by robust national governance, policy briefs,^10^ and regular dialogue with the private sector. The PPM partners have developed models for mapping, training, supportive supervision, and monitoring private providers across the country. These models operate within hubs (private for-profit facilities proving full range of TB services) and spoke frameworks, with community-based providers serving as spokes: drug-venders and informal community-based providers play a pivotal role in TB screening and referral within the PR-supported area. In 2022, the private sector contributed 24% of the country’s TB notifications, with over 24,000 informal providers accounting for 55% of private sector notifications. Collaborative efforts between the Global Fund, USAID-funded projects, and the NTP have facilitated the implementation and expansion of PPM activities nationwide. Despite the challenges posed by the COVID-19 pandemic, Nigeria’s TB response demonstrated resilience, with private providers making a significant contribution.^11,12^ Private provider notifications increased from 13,031 in 2014 to 69,504 in 2022,^13^, playing a crucial role in narrowing the gap between overall TB notifications and estimated incidence.

In the Philippines, ongoing efforts by the Department of Health, PRs and SRs are raising awareness among registered physicians about the mandatory notification law. In addition, private providers and their patients benefit from referral arrangements for TB screening and testing at nearby facilities and access to mobile applications to facilitate notifications and treatment follow-up. In 2019, mandatory notification supported through dedicated notification officers accounted for 90% of all private sector notifications.^9^ To enhance access to screening and testing services for economically disadvantaged patients attending private facilities, a free voucher known as “RxPass” is provided through general practitioners (GPs). By 2023, 29% of GPs used the RxPass. Additionally, motorcycle riders (called “STRiders”) transport sputum samples from private and public providers/facilities to laboratories for testing. In 2022, the private sector contributed 21% of TB case notifications, with improvements seen in the utilisation of GeneXpert testing among notified patients (from 18% in January 2022 to 31% by July 2022). The new grant (GC7) will intensify support for improved access to screening/testing services, including enhancing the quality of bacteriological confirmation and treatment outcomes.

Pakistan has significantly scaled up its engagement with the private sector in its national TB programme over the past several years. Private sector contributions to TB notifications have increased from 33% in 2018 to 42% in 2022. GPs record the largest contributions, with notifications surging from 26,000 in 2015 to approximately 134,000 in 2022.^14^ Overall, the private sector contributed 176,151 TB cases in 2022.^15^ The private sector has consistently maintained a TB treatment success rate exceeding 90% over the past several years. However, the proportion of clinically diagnosed TB remains high primarily due to limited access to GeneXpert testing and an overreliance on X-ray findings. To address these issues, Global Fund grants are facilitating the provision of GeneXpert machines to the private sector and expanding sample transportation.

### Trends in TB and PPM budgets for the seven priority countries, 2002–2026

**Figure 1.**
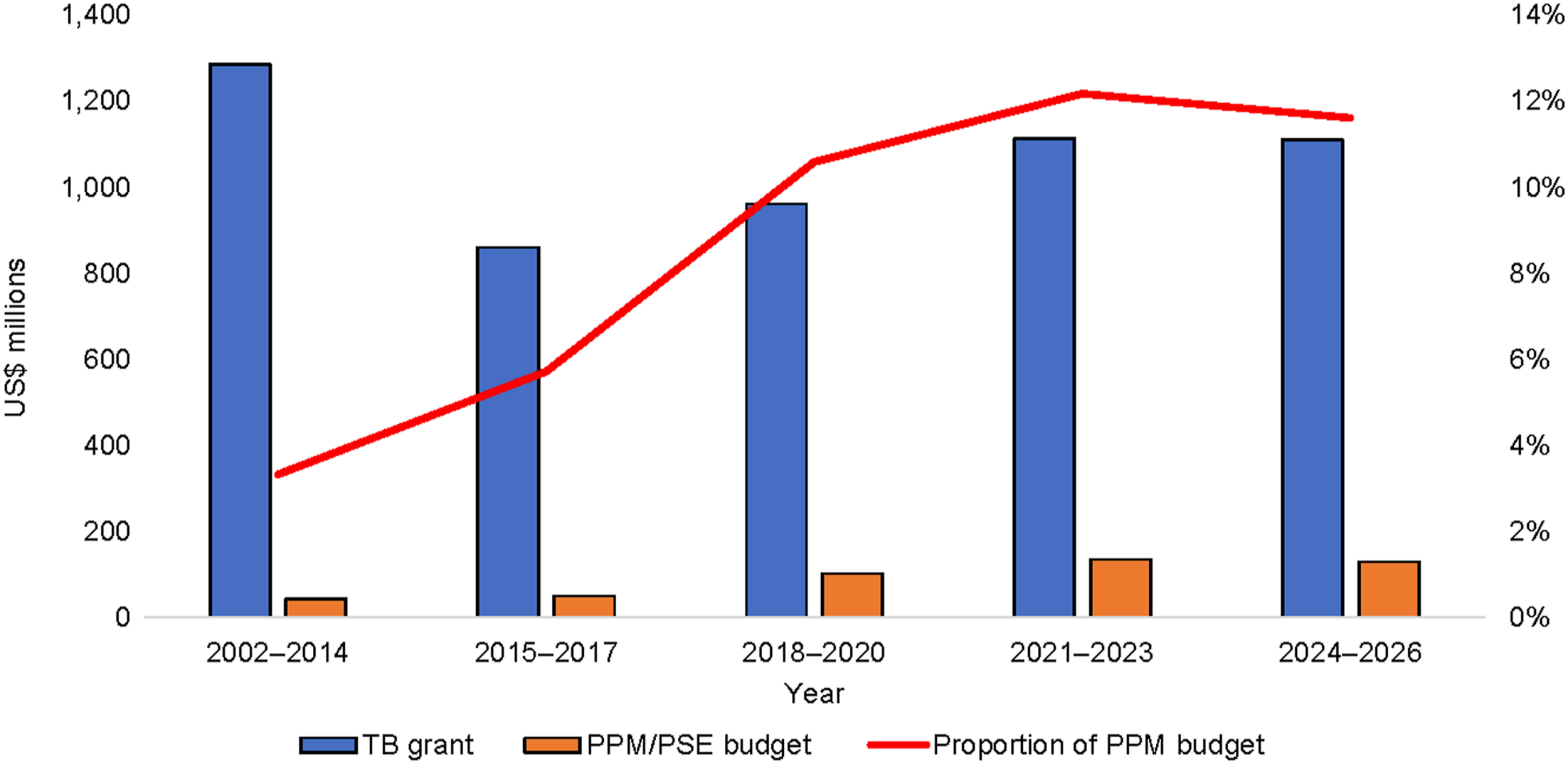
Trends in total TB and PPM budget in Global Fund grants in select countries (Bangladesh, India, Indonesia, Myanmar, Nigeria, Pakistan, The Philippines), 2002–2026. PPM = private-public mix; PSE = private sector engagement.

**Figure 2.**
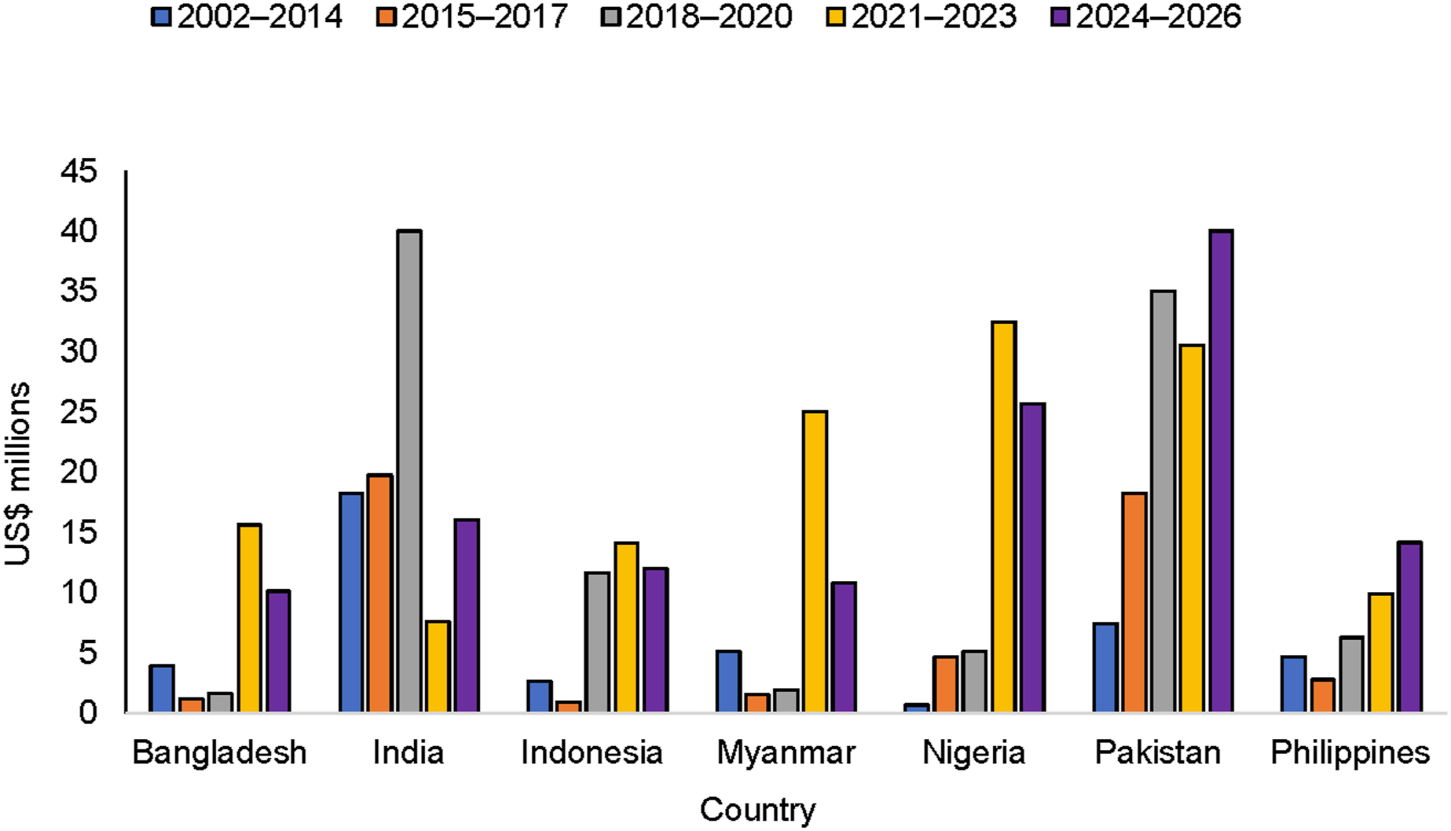
Trends in PPM budget. PPM = private-public mix.

In general, the TB and PPM budgets increased over time (Figures 1 and 2). The proportion of budget allocated for PPM increased from 3% during the Global Fund’s rounds-based funding model (2002–2014) to 6% in 2015–2017, 11% in 2018–2020, and 12% in 2021–2023 and 2024–2026, respectively.

Figure 2 shows trends in the PPM budget for each country. In most of the countries reviewed, the PPM budget has increased over time. For India, the largest PPM budget was allocated during GC5 when the PPSA model was implemented by three NGO PRs (JEET), which was transitioned to domestic funding (NTEP) during GC6; hence, the budget was reduced in the Global Fund grants. The reduction in the PPM budget for some countries was mainly due to changes in the way the PPM budget was categorised in the Global Fund framework.

To ensure the availability and quality of data on private sector engagement (financial and programmatic), the Global Fund has created a new module (‘collaboration with all sectors’, and a new intervention called ‘private sector engagement in TB’) in the modular framework for GC7. Additional indicators have been incorporated in the performance framework (PF) to track private sector contributions to TB response beyond ‘notification’, including the disaggregation of notifications by bacteriologically confirmed and clinically diagnosed cases, as well as tracking treatment outcomes. Ten of the 11 countries have included the indicator on notifications and nine of the countries have included the indicator on treatment outcomes in their PF for GC7. These changes will allow for improved tracking, not only in coverage but also in the quality of TB services provided by private providers.

## DISCUSSION

The Global Fund’s financial support for the private sector has increased over time, creating an opportunity to engage more private stakeholders and providers in managing grants and implementing TB activities along the cascade of care. However, the budget remains small compared with the PPM contributions to TB notifications. There is no correlation between the PPM budget and its contribution to notification targets. In Myanmar, the two proportions are similar, and in Vietnam, the proportion of the PPM budget is higher than the PPM contribution to notification, with a wide variation in other countries. This could be due to different reasons, including the fact that the PPM budget in our analysis is from the Global Fund grants only, while the PPM notification targets are national and could be supported through other funding sources as well. Obtaining notification data from the private sector could require less resources as patients bear the costs of accessing TB services from private providers. It is also important to note that proportional funding increase alone is not sufficient to explain success, and further comprehensive analysis is warranted. Although the overall TB budget for the 11 countries from the Global Fund has been increasing, the countries have different levels of TB burden, health system, domestic funding available for TB, and high-level commitment. The performance of the national TB notification targets and the contribution of PPM could be affected by multiple factors beyond funding availability. Private healthcare providers are important partners for ensuring the provision of care to all people wherever and when they seek care. As many individuals with TB symptoms initially seek care from private providers, their engagement is essential for effective TB response.^16^

Although making TB a notifiable disease and establishing mandatory notification policies are critical first steps undertaken in all 11 countries, operationalisation and enforcement of these policies require additional commitments and resources. This includes incentivising private healthcare providers to promptly notify people diagnosed with TB.^17^ In Pakistan, effective implementation of the law requires training healthcare providers on TB case management guidelines, strengthening district health authorities to ensure persuasive enforcement of the law, and involving healthcare commissions in the enforcement process.^18^ In Indonesia, a study highlighted the recognition of mandatory TB notification’s importance among private practitioners.^19^ This acknowledgement was linked to enhanced case detection and reduced treatment dropout. However, the challenges in enforcing the law include private providers’ limited knowledge of the policy and concerns about potential penalties. User-friendly approaches such as SMS text messages, phone calls, or online applications could facilitate effective implementation of mandatory notification laws/policies.^19^

Since 2012, the Government of India has enforced a mandatory TB notification policy, resulting in a consistent increase in TB notifications from the private sector. Private sector notifications have surged more than sevenfold since 2014, constituting over 30% of total notifications to date.^20^ However, limited awareness of the electronic notification methods and a preference for paper-based reporting among private providers were identified in one State as key areas for improvement in order to successfully transition from a paper-based to an electronic notification system.^21^ These challenges have been addressed by optimising the electronic recording and reporting system (called *Ni-kshay*), which is now available for private providers with support from intermediary agencies.

As shown in our analysis, private providers have contributed to a quick recovery in TB response during and after the COVID-19 pandemic, further highlighting the need to work with all stakeholders and partners. A rapid assessment of intermediary NGOs in seven high TB burden countries reported disruptions caused by COVID-19 and how the private sector, despite these challenges, demonstrated resilience and adaptability and ensured continued provision of TB services.^22^

The momentum created as part of finding missing people with TB provided an opportunity to accelerate engagement with all providers.^23^ The catalytic/matching funds in GC7 continue to focus on ‘finding the missing people with TB/DR-TB and successfully treating them’ in 20 priority countries, including all 11 countries in our analysis. This means that additional resources will be available to further strengthen private sector engagement and accelerate the uptake and scale-up of tools (existing and new) and innovative models. Involving private actors not only has a major impact on TB response but also contributes to the prevention of drug resistance.

When implementing innovative PPM or private sector engagement (PSE) models, countries should be flexible in adopting lessons learned and experience both locally and internationally. Our analysis highlights numerous successful examples of the role of private entities in TB response supported by the Global Fund and others. Furthermore, direct correlations have been observed between the effectiveness of TB response and the level of engagement of private actors along the TB cascade of care.^24,25^

Despite the significant enhancement of PPM through the support of the Global Fund and others, there is a need to transition such models to domestic funding, reduce dependence on donor funding, and enhance sustainability. India stands out as an excellent example, having substantially increased funding for PSE in TB responses in its domestic budget in recent years, including supporting the PPSA model through domestic funding and providing nutritional support to all TB patients.

Furthermore, the significance of Social Health Insurance (SHI) in sustaining PSE cannot be overlooked. In countries such as Indonesia and the Philippines, SHI schemes are progressing towards comprehensive coverage for the entire population. The ongoing expansion of SHI and the broader Universal Health Coverage (UHC) movement are concurrently fostering awareness about the importance of involving private healthcare providers to attain UHC.^5^ It is critical that SHI expands its focus to engage primary-level private healthcare providers while strengthening high-volume private hospitals so that people-centred services are available everywhere.^5^

The Global Fund Strategy sets out the opportunity for new partnerships aimed at reaching the 2030 goals for TB and advocates for evidence-based planning and the scale-up of innovative and integrated people-centred approaches.^26^ Our analysis highlights the need for and opportunities to scale up engagement of private providers along the TB cascade of care and the importance of allocating proportional budget for PSE in TB grants as well as catalysing additional funding from domestic and other sources, which agree with the PPM Roadmap.^5^ Governments and all stakeholders are responsible for creating a healthy, well-balanced environment for effective collaboration between public/private entities in public health: a strong partnership to prevent and combat diseases.

Our analysis may not fully capture the budget for PPM as procurement of essential items for patients diagnosed and/or treated by private healthcare providers is typically done through NTPs and is not included in the PPM budget. Moreover, financial support for PPM activities from other sources, including domestic funding, was not captured in our analysis, while the private sector TB notification encompasses all providers irrespective of the source of financial support. Despite these limitations, our analysis provides important information on the Global Fund’s investments in TB and PSE, and how these investments catalyse and help countries to accelerate progress towards ending TB.

## CONCLUSION

Our indicative analysis underscores successful endeavours across various countries, showcasing the pivotal role of private healthcare providers in TB response. Evidence-based planning and increased investment from domestic budgets, the Global Fund, and other sources in private sector involvement and a multifaceted partnership and collaboration between the public, private sector and communities are indispensable to end TB.
